# Ceramic Embedded Junction Thermocouple for In Situ Transient Temperature Measurement in Confined Extreme Thermal Environments

**DOI:** 10.3390/mi17101163

**Published:** 2026-10-03

**Authors:** Mingfeng E, Jingyue Fan, Yuxin Song, Fengyuan Ma, Junwei Yin, Yunxian Cui, Jun Deng

**Affiliations:** School of Mechanical Engineering, Dalian Jiaotong University, Dalian 116028, China; emf531895757@126.com (M.E.); 15985179848@163.com (J.F.); 18434757786@163.com (Y.S.); mafengyuan198@163.com (F.M.); yjwzx@djtu.edu.cn (J.Y.); dlcyx007@126.com (Y.C.)

**Keywords:** embedded junction thermocouple, transient temperature measurement, confined explosion environment, ceramic substrate, dynamic response

## Abstract

Transient temperature measurement under confined extreme thermal environments requires sensors with rapid response and structural stability. Conventional thermocouples often suffer from delayed response or insufficient junction protection, leading to inaccurate characterization of local thermal loads. In this work, a ceramic embedded junction thermocouple was developed for in situ transient temperature measurement. The sensor structure was optimized by finite element simulation, and a NiCr/NiSi junction was embedded into an Al_2_O_3_ ceramic substrate through inorganic bonding, thermal treatment, and surface grinding. The fabricated sensor was evaluated through thermoelectric characterization, high-temperature stability testing, dynamic response measurement, and calibration. The sensor exhibited a Seebeck coefficient of 41.26 μV/°C from 300 to 1200 °C, maintained continuous output at 1200 °C for 8 h, and achieved a response time of approximately 187 μs. The maximum relative deviation was below 0.47% within 300–1000 °C. During a confined thermal nose explosion test, the sensor successfully captured transient temperature evolution and recorded a peak temperature of approximately 1289 °C. Conventional thermocouples recorded peak temperatures below 800 °C, which were approximately 40% lower than the developed sensor. The proposed sensor provides a reliable approach for localized transient temperature measurement under confined extreme thermal environments.

## 1. Introduction

Explosions involve an intense release of energy within a very short period and can generate rapidly varying environments with high-temperature, high pressure, and strong impact in confined spaces [1,2,3]. The local temperature after an explosion reflects both the energy release and the associated heat transfer processes, and it is also closely related to the thermal loads imposed on surrounding structures [4,5,6]. Therefore, transient temperature measurements at specific locations are important for analyzing explosion thermal effects, designing thermal protection structures, and evaluating structural safety [7,8,9,10]. Unlike steady state high-temperature measurements, temperature fields in explosion environments are characterized by short durations, rapid variations, and pronounced spatial nonuniformity [11,12,13]. These characteristics place stringent requirements on the temporal response and environmental adaptability of temperature measurement systems [14,15]. Failure to capture transient temperature peaks and their temporal evolution may lead to inaccurate characterization of explosion thermal loads and subsequent structural thermal responses [16,17]. Consequently, transient temperature sensors capable of stable operation in harsh explosion environments are essential for explosion-related thermal measurements. Related studies on high-temperature thin film sensors have also shown that material selection and structural design are important for maintaining sensing performance under severe thermal conditions [18,19].

Temperature measurements in explosion environments are mainly performed using noncontact optical techniques or contact thermocouples. Optical methods based on two color pyrometry, high speed imaging, and multispectral radiation have been applied to transient temperature measurements of explosion fireballs [20,21,22]. These methods can provide temporal or spatial temperature information without direct contact with the explosion products. However, an effective optical path between the target region and the detection system is required. Their applicability is therefore limited when the measurement location is inside a confined cavity, obscured by surrounding structures, or inaccessible through a direct observation window. For measurements requiring the sensing element to be positioned directly at a specified location, thermocouples remain attractive because they require no external excitation, provide a wide temperature range, and can be readily configured as insertion probes for in situ local temperature measurements.

Reducing the dimensions and thermal mass of the thermocouple sensing region is an effective approach for improving its ability to follow rapid temperature variations [23]. Liu et al. [24] developed chromel–nickel thin film thermocouples and reported a response delay of approximately 75 μs during step temperature calibration, demonstrating the rapid response enabled by a sensing structure with low thermal mass. Wang et al. [25] fabricated a Pt-Rh10/Pt thin film thermocouple on an Al_2_O_3_ ceramic substrate and evaluated its dynamic response using pulsed laser calibration, obtaining a time constant of approximately 535 μs. This study provides relevant evidence that ceramic substrates can be integrated with thermocouple sensing structures while maintaining sub-millisecond dynamic response. However, when thermocouples are directly exposed to explosion environments, reducing the dimensions of the sensing element also raises concerns regarding structural integrity. Frost et al. [26] used K-type thermocouples with different wire diameters for in situ temperature measurements in multiphase fireballs generated by metallized explosives. They found that thinner thermocouples responded more rapidly to temperature variations but were less likely to remain intact throughout the complete explosion event. Thus, simply reducing the dimensions of the thermoelectric wires and junction to minimize thermal inertia is not sufficient, because structural stability must also be considered under explosion loading. To improve the structural stability of thermocouples used in confined explosion environments, Niu et al. [27] developed a Type C thermocouple with W5%Re/W26%Re alloy strips as the sensing elements and an Al_2_O_3_ ceramic structure for probe fixation. They indicated that the thickness of the sensing element must be balanced between mechanical robustness and response time. An alloy strip with a thickness of 0.1 mm was ultimately selected, resulting in an average response time of approximately 13 ms during laser step calibration. At this response level, transient temperature changes on the microsecond scale cannot be fully captured without dynamic distortion. These studies indicate that, for thermocouple probes inserted directly into confined explosion environments, an important structural challenge is to provide sufficient support for the sensing junction while maintaining a small effective sensing region.

To address this challenge, a thermocouple with a ceramic substrate and an embedded junction was developed in this study for transient temperature measurement in explosion environments. Finite element analysis was first used to determine the overall sensor dimensions and design the armored structure. A NiCr/NiSi thermocouple junction was formed by welding and locally embedded in a groove at the front end of an Al_2_O_3_ ceramic substrate through inorganic bonding and thermal treatment. The sensing end was subsequently ground and polished to form the final sensing region. A mechanical connection between the ceramic substrate and the nickel alloy armored shell was also designed. Together with a threaded mounting section and rear lead protection, these components formed a complete insertion probe. The fabricated sensor was systematically evaluated in terms of its thermoelectric characteristics, dynamic response, high-temperature stability, and measurement accuracy. Finally, the sensor was applied in a nose cavity explosion test to evaluate its feasibility for local transient temperature measurements in confined explosion environments.

## 2. Materials and Methods

### 2.1. Working Principle of the Thermocouple Sensor

A thermocouple measures temperature based on the thermoelectric effect [28]. When two dissimilar conductive materials form a closed circuit and their two junctions are maintained at different temperatures, a thermoelectric voltage is generated. The temperature difference can therefore be determined from the measured voltage. The thermoelectric voltage $E_{\mathrm{AB}}\left(T,T_{0}\right)$ can be expressed as the integral of the difference between the Seebeck coefficients of the two materials over the corresponding temperature range:

(1) $$E_{\mathrm{AB}}\left(T,T_{0}\right)=\int_{T_{0}}^{T}\left(S_{A}-S_{B}\right)dT$$

where $S_{A}\;$ and $S_{B}$ are the Seebeck coefficients of thermoelectric materials A and B, respectively. T is the hot junction temperature, and $T_{0}$ is the cold junction temperature. If the cold junction temperature is maintained at a fixed value and the Seebeck coefficients are treated as constants over the relevant temperature range, the hot junction temperature can be written as:

(2) $$T=\frac{E_{\mathrm{AB}}}{S_{A}-S_{B}}+T_{0}$$

Based on this measurement principle, the thermocouple sensor was further designed for the high temperature, impact loading, and limited installation space encountered in explosion environments. The design focused on material selection, overall dimensions, the ceramic substrate, the embedded junction, and the armored shell.

### 2.2. Structural Design of the Thermocouple Sensor

The sensor developed for temperature measurement in the nose cavity during explosion tests was designed as an insertion probe. It mainly consists of thermoelectrodes, an Al_2_O_3_ ceramic substrate, an armored shell, and a rear lead protection structure. The materials were first selected according to the functional requirements of each component. The overall dimensions were then determined based on the available installation space in the nose cavity and the structural response of the probe. On this basis, the ceramic substrate, embedded junction, armored shell, and mounting structure were designed.

#### 2.2.1. Material Selection

The thermoelectrodes, a ceramic substrate, and an armored shell are responsible for temperature sensing, electrical insulation and structural support, and overall mechanical protection, respectively. Material selection therefore needs to consider high-temperature stability, electrical properties, and mechanical reliability. NiCr and NiSi were selected as the thermoelectric materials to form a Type K thermocouple. NiCr/NiSi thermocouples provide stable thermoelectric output and good repeatability, together with suitable oxidation resistance at high temperatures [29]. They also have a substantially lower material cost than noble metal thermocouples. These characteristics make the NiCr/NiSi combination suitable for transient temperature measurements in explosion environments where both sensitivity and high-temperature capability are required. High purity Al_2_O_3_ ceramic was selected as the substrate material. Al_2_O_3_ provides high electrical insulation at elevated temperatures, together with high mechanical strength, hardness, thermal stability, and chemical stability [30]. It can therefore maintain electrical isolation between the two thermoelectrodes while providing structural support for the thermoelectrodes and the sensing junction. In the embedded junction configuration developed in this study, the ceramic substrate also performs several structural functions, including thermoelectrode positioning, local junction constraint, and mechanical engagement with the armored shell. High purity Al_2_O_3_ was therefore selected as the substrate material. A high-temperature nickel alloy was used for the armored shell. Nickel alloys provide high-temperature strength, oxidation resistance, and corrosion resistance, allowing the sheath to maintain structural stability under elevated temperature conditions [31]. In the present sensor, the armored shell protects the internal ceramic substrate and thermoelectrodes and also provides the mechanical interface between the sensor and the test structure. The armored shell material must therefore satisfy both thermal and mechanical requirements. Considering the intended explosion environment, a high-temperature nickel alloy was selected for the sensor armored shell.

#### 2.2.2. Overall Dimension Design

The sensor was developed for local in situ transient temperature measurement in confined explosion spaces, with the nose cavity of a target projectile selected as the specific application and structural design case. The probe is inserted into the cavity through a predefined mounting interface so that the sensing end reaches the intended measurement region. The probe length was determined by the location of the target measurement region. According to the geometry of the nose cavity and the sensor mounting position, an insertion depth of approximately 50 mm allows the sensing end to reach the required location. Taking the mounting structure into account, the total sensor length was set to 55 mm. Once the length was determined by the measurement position and installation conditions, the outer diameter became the main geometric parameter affecting both the overall probe size and its structural response in the cantilevered state.

After installation, part of the sensor extends into the nose cavity. A smaller outer diameter reduces the bending stiffness of this unsupported section and can result in larger deformation and stress under external flow loading. Increasing the diameter improves structural stability but also increases the sensor volume and is constrained by the available space inside the cavity. Therefore, five sensor models with outer diameters of 4, 6, 8, 10, and 12 mm were established while maintaining the same total length. Their structural responses were then compared numerically.

**Figure 1:**
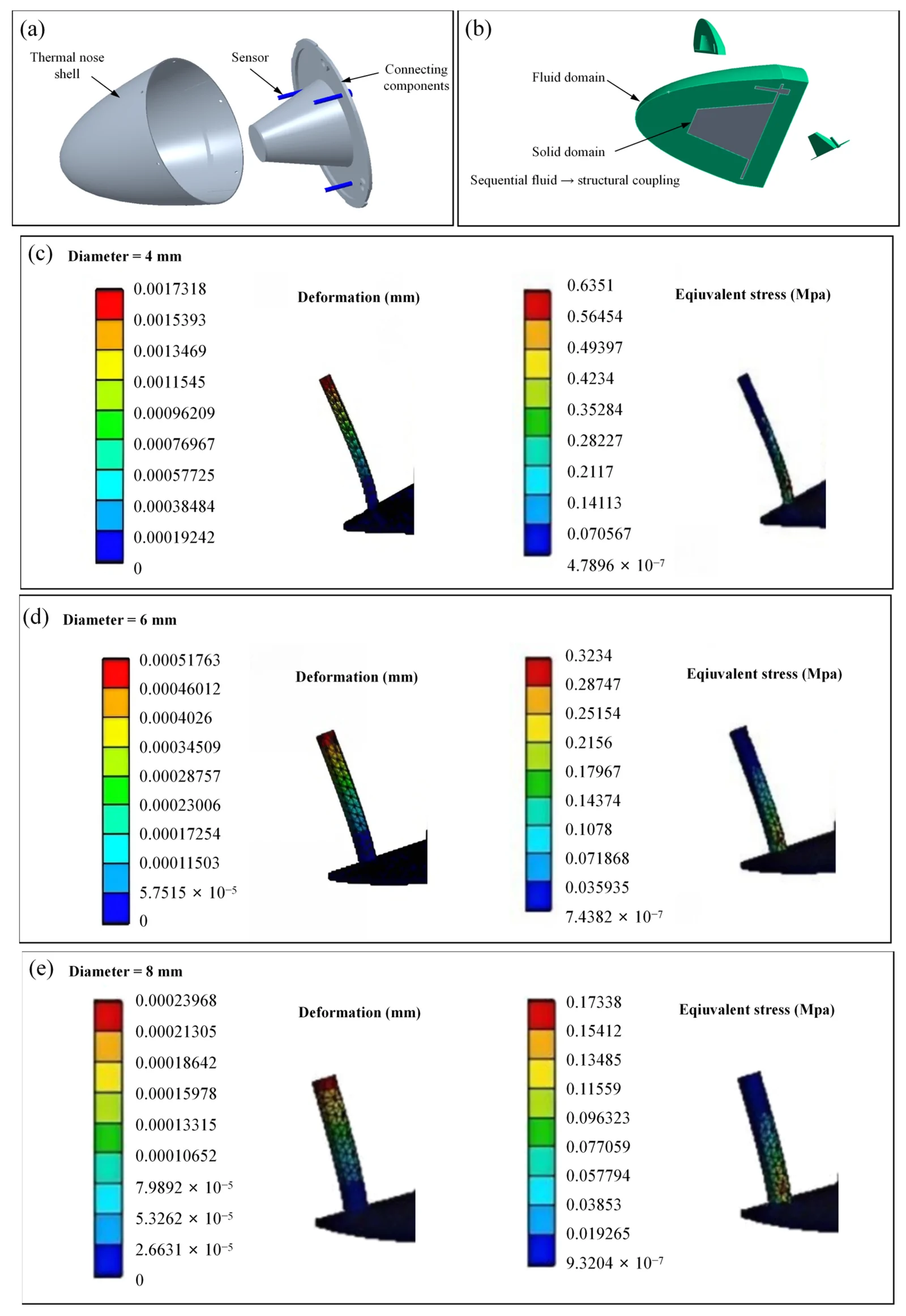

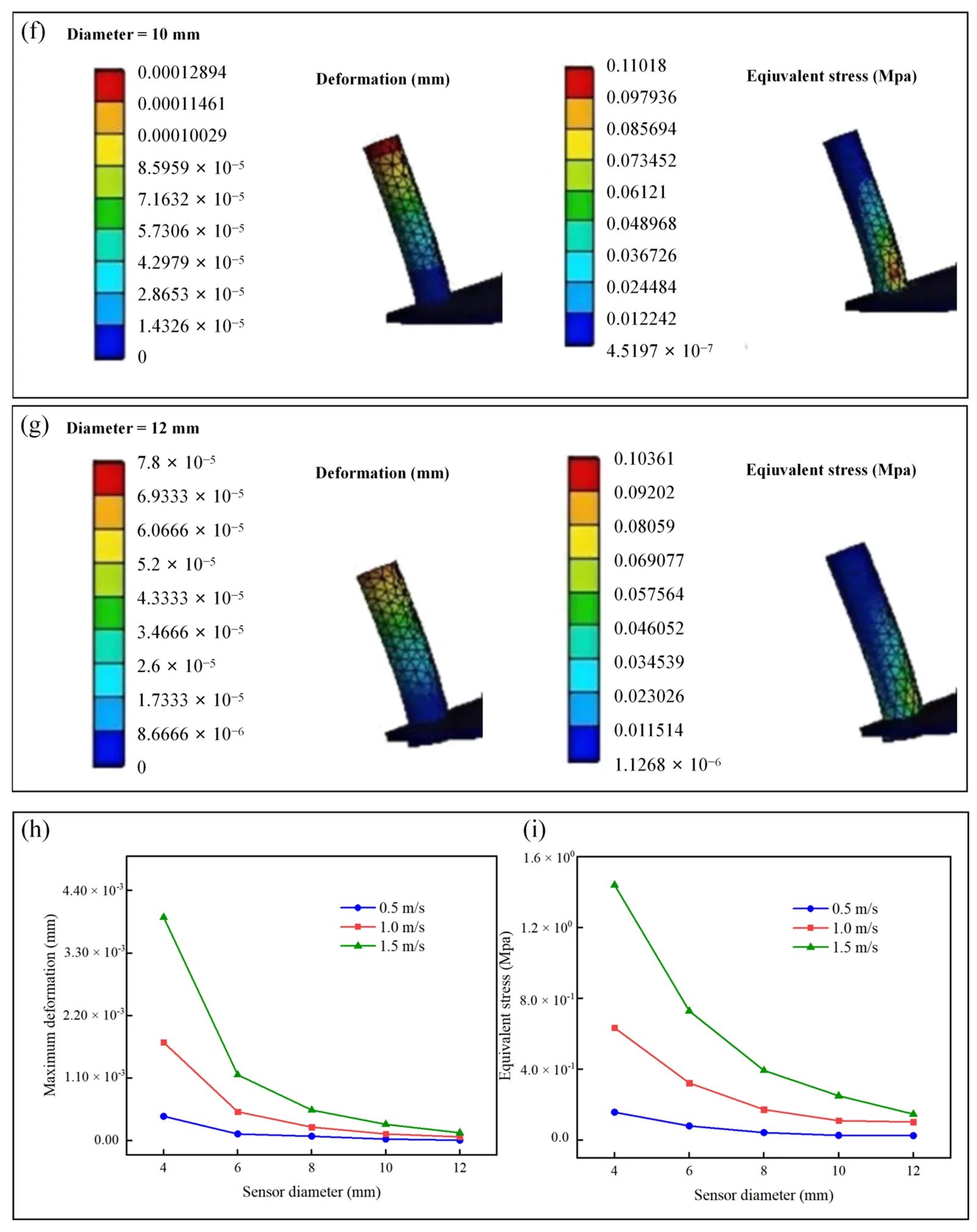
Structural design and flow–solid coupling simulation of the thermal nose sensor assembly. (**a**) Three-dimensional model of the thermal nose structure and sensor assembly. (**b**) Computational domain for one-way fluid–solid coupling simulation. (**c**–**g**) Simulated deformation and equivalent stress distributions of sensor assemblies with different diameters under a flow velocity of 1.0 m/s: (**c**) 4 mm, (**d**) 6 mm, (**e**) 8 mm, (**f**) 10 mm, and (**g**) 12 mm. (**h**) Maximum deformation variation with sensor diameter under different flow velocities. (**i**) Maximum equivalent stress variation with sensor diameter under different flow velocities.

A one-way fluid and structural coupling approach was used to establish the numerical model of the sensor and the local nose cavity structure. Because the cavity is geometrically symmetric, only half of the structure was modeled to reduce computational cost. The sensor installation configuration and computational domain are shown in Figure 1a,b. To compare the relative structural responses of sensors with different outer diameters under consistent fluid loading, three nominal inlet velocities of 0.5, 1.0, and 1.5 m/s were selected as low, intermediate, and high loading levels. These conditions were used for parametric comparison rather than for reproducing the transient blast wave. The flow field was calculated first, and the resulting surface loads were subsequently transferred to the structural model. Maximum deformation and equivalent stress were selected as the evaluation parameters. The responses of sensors with different outer diameters were compared to provide a basis for selecting the overall sensor dimensions.

The deformation and equivalent stress distributions obtained at an air velocity of 1.0 m/s are presented in Figure 1c–g as representative simulation results. The complete numerical results for all simulated conditions are summarized in Table 1.

As can be seen from Table 1, Figure 1h,i, for all three flow velocity conditions, both the maximum deformation and the equivalent stress decrease as the sensor outer diameter increases. This trend indicates that a larger outer diameter improves the structural stiffness of the cantilevered probe under the same flow loading. At 1.0 m/s, increasing the outer diameter from 4 to 8 mm reduced the maximum deformation from 1.73 × 10^−3^ to 2.4 × 10^−4^ mm and the equivalent stress from 0.6351 to 0.1733 MPa, corresponding to reductions of approximately 86.1% and 72.7%, respectively. Further increases in diameter to 10 and 12 mm continued to reduce both parameters. However, larger diameters also increased the transverse dimensions of the probe and reduced the space available for the ceramic substrate, armored shell, and mounting interface. Considering the available space inside the nose cavity, the structural response of the probe, and the requirements for subsequent fabrication and assembly, the final sensor dimensions were selected as 8 mm in outer diameter and 55 mm in total length.

#### 2.2.3. Design of the Ceramic Substrate and Embedded Sensing End

**Figure 2:**
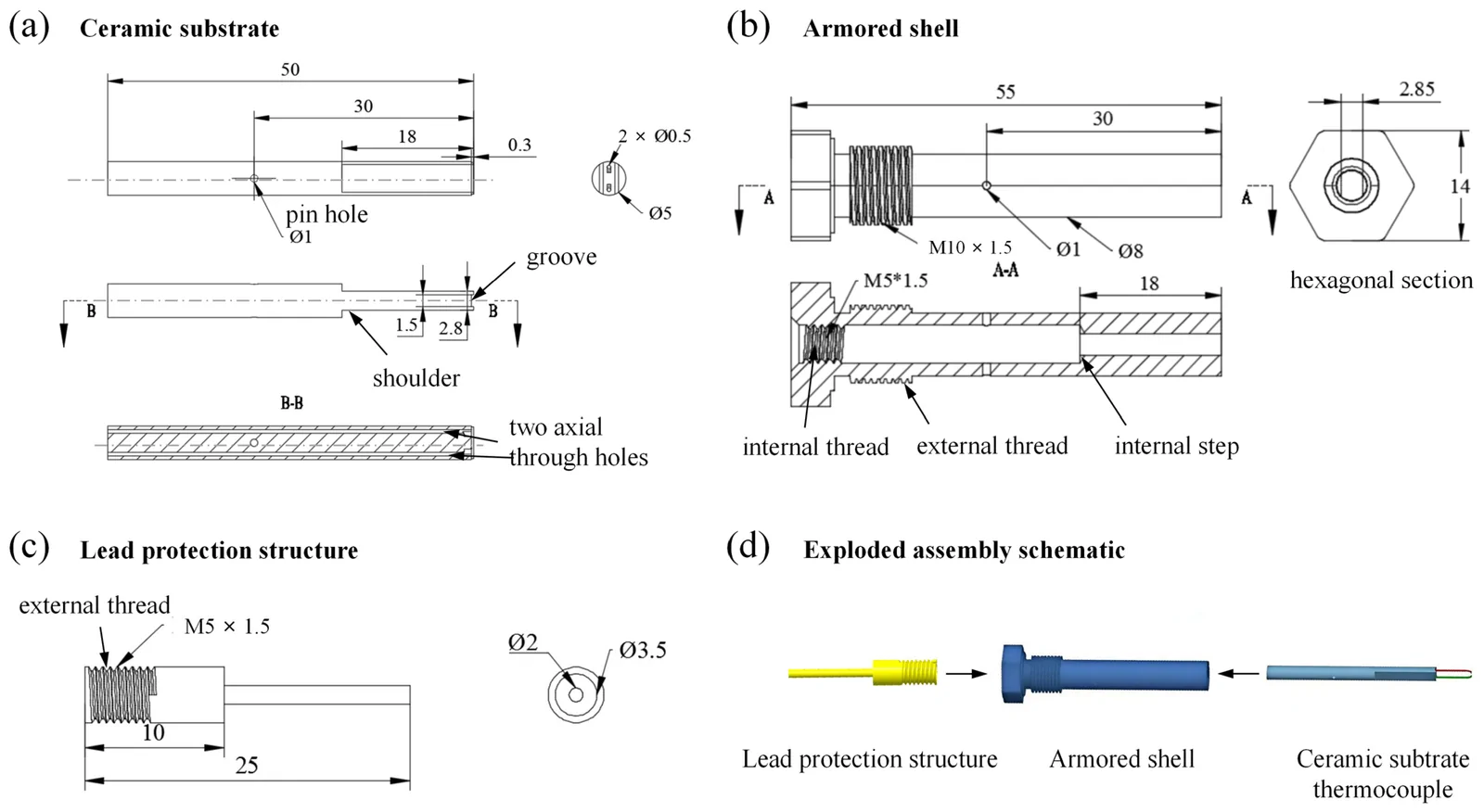
Structural design and assembly of the ceramic embedded junction thermocouple sensor. (**a**) Al_2_O_3_ ceramic substrate. (**b**) Nickel alloy armored shell. (**c**) Lead protection structure. (**d**) Exploded assembly configuration of the sensor.

After the overall sensor dimensions were determined, the ceramic substrate and sensing end were designed, as shown in Figure 2a. The substrate provides electrical insulation and positional constraint for the thermoelectrodes and also serves as the structural carrier between the sensing junction and the armored shell. Considering the selected sensor outer diameter of 8 mm and the wall thickness required for the armored shell, the ceramic substrate was designed as a cylindrical component with a diameter of 5 mm and a length of 50 mm. Two axial through holes with a diameter of 0.5 mm were machined in the ceramic substrate to accommodate the 0.3 mm NiCr and NiSi thermoelectrodes. The two holes provide independent paths for the thermoelectrodes and maintain electrical isolation through the surrounding Al_2_O_3_ ceramic. They also constrain the lateral movement of the wires and guide them toward the front sensing end.

For a conventional wire thermocouple, the welded junction is located at the ends of the two thermoelectrodes. When such a junction is directly inserted into an explosion environment, it lacks local structural support and may be susceptible to mechanical damage. To address this issue, a shallow recess was introduced at the front end of the ceramic substrate to accommodate the NiCr/NiSi junction. Based on the measured size of the welded junction, the recess was designed with a diameter of 1 mm and a depth of 0.3 mm. This geometry allows the junction to be positioned within the front surface of the ceramic substrate. Rather than completely covering the junction with an external protective layer, the design provides local ceramic support around the junction and forms the geometric basis for the subsequent embedded sensing end.

The ceramic substrate was also designed to form a stable mechanical connection with the armored shell. An 18 mm long shoulder was introduced on the substrate, and a corresponding internal step was machined into the armored shell. After assembly, the shoulder engages with the internal step to provide axial positioning and support. A 1 mm diameter pin hole was further machined through the corresponding positions of the ceramic substrate and armored shell. A cylindrical pin was inserted during assembly. The pin restricts relative rotation and axial displacement between the two components. Together with the substrate shoulder and the internal step of the armored shell, it forms the mechanical positioning structure of the ceramic substrate. This arrangement also allows the individual components to be fabricated separately and assembled subsequently.

The sensing end is the key functional region of the ceramic substrate. After the NiCr and NiSi thermoelectrodes are passed through the axial holes, the welded junction is positioned inside the front recess and bonded to the ceramic substrate using the subsequent embedding process. The front surface is then machined so that the junction is formed at the sensing surface. As a result, the junction is no longer suspended outside the substrate as an unsupported welded bead. Instead, the surrounding ceramic provides local support to the region adjacent to the junction while the sensing surface remains directly exposed to the thermal environment.

The junction size is an important factor affecting the dynamic response of a thermocouple. For the same material system, an increase in junction size increases the effective thermal mass of the sensing end and can prolong its temperature response. Therefore, the effective junction size must be controlled during final machining while maintaining electrical continuity and structural integrity. The resulting ceramic substrate integrates several functions, including electrical insulation and positioning of the thermoelectrodes, local embedding of the sensing junction, axial positioning of the substrate, and mechanical connection to the armored shell. This integrated structure provides the basis for subsequent assembly of the complete sensor.

#### 2.2.4. Design of the Armored Shell and Lead Protection Structure

After the ceramic substrate and embedded sensing end were defined, the armored shell, mounting interface, and rear lead protection structure were designed, as shown in Figure 2b,c. For an insertion probe used in explosion environments, the armored shell must provide mechanical support for the ceramic substrate and thermoelectrodes while leaving the front sensing region unobstructed. Therefore, an open front nickel alloy armored shell was adopted. This arrangement places the ceramic sensing surface at the front of the armored shell and allows it to exchange heat directly with the surrounding environment.

Based on the overall dimension design described above, the sensor body diameter was fixed at 8 mm. The internal dimensions of the armored shell were matched to the 5 mm diameter ceramic substrate. An internal step corresponding to the substrate shoulder was machined into the armored shell to provide axial positioning. A 1 mm diameter pin hole was also machined through the side wall of the armored shell and aligned with the corresponding hole in the ceramic substrate. During assembly, a cylindrical pin mechanically connects the two components and restricts relative rotation and axial displacement.

To enable in situ installation in the nose cavity, an external thread and a hexagonal section were machined on the sheath. An M10 × 1.5 thread was selected for connection between the sensor and the experimental structure, while the hexagonal section facilitates installation and removal. An internal thread was machined at the rear of the sheath and matched with the external thread of the lead protection tube. This design allows the sensing body and rear protection structure to be fabricated separately and assembled afterward. The rear lead protection tube was designed with a stepped geometry to provide mechanical protection for the thermoelectrodes and connecting leads. The stepped structure also helps limit the ingress of external particles and contaminants into the sensor. The exploded assembly schematics are shown in Figure 2d.

After the main protective structure was established, the thermal protection requirement of the rear lead region was further considered. During high-temperature exposure, heat is transferred from the sensing end toward the rear region through the NiCr/NiSi thermoelectrodes, Al_2_O_3_ ceramic substrate, and nickel alloy armored shell. To evaluate the axial temperature distribution and determine the thermal exposure of the rear leads, a transient heat transfer model of the complete sensor was established. The thermal properties of NiCr, NiSi, Al_2_O_3_ ceramic, and nickel alloy were assigned according to their corresponding material characteristics. The thermal conductivity, specific heat capacity, and density used in the simulation are summarized in Table 2.

**Figure 3:**
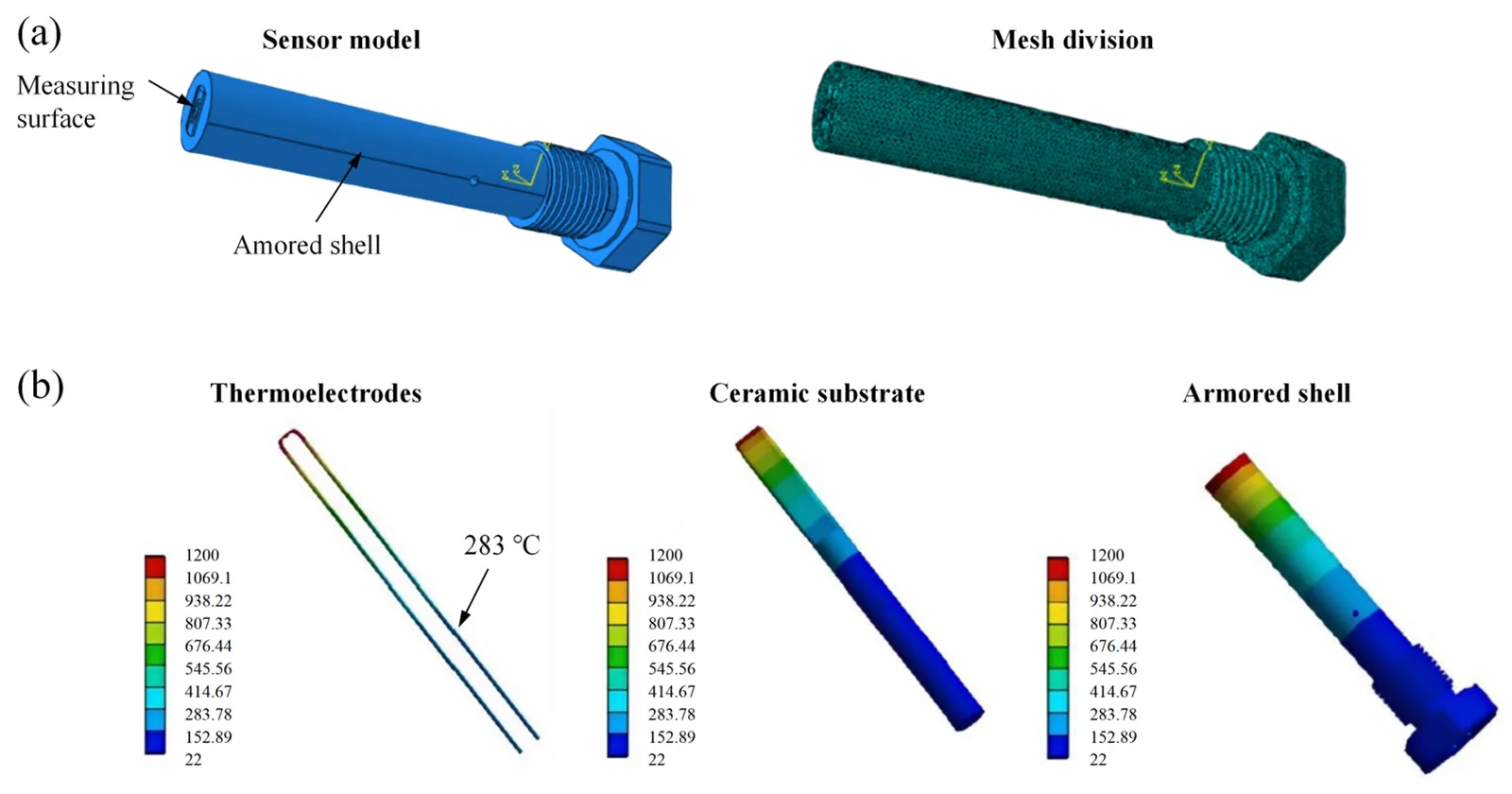
Sensor heat transfer simulation temperature isothermal surface. (**a**) Model of the sensor. (**b**) The temperature distributions of the thermoelectrodes, ceramic substrate, and armored shell.

A temperature boundary of 1200 °C was applied to the sensing end for 60 s to simulate a high-temperature transient exposure condition. All other external surfaces were defined as thermally insulated boundaries. The simulation model of the sensor is shown in Figure 3a. The temperature distributions of the thermoelectrodes, ceramic substrate, and armored shell are shown in Figure 3b. The simulation results indicate that after exposure to the 1200 °C thermal load for 60 s, the temperature at the rear section of the 60 mm long thermoelectrodes remained within 152–283 °C. This result provides a reference for selecting the rear lead protection component. Therefore, a protection tube capable of continuous operation above 300 °C was selected to protect the thermoelectrodes and ensure reliable operation of the sensor under elevated temperature conditions.

After the above structural design, the complete sensor consists of an embedded ceramic sensing end, nickel alloy armored shell, mechanical positioning structure, and rear lead protection assembly. This integrated design provides the structural basis for subsequent fabrication and assembly.

### 2.3. Fabrication of the Ceramic Embedded Junction Thermocouple

After completing the structural design, the thermocouple sensor was fabricated following the sequence of junction preparation, ceramic substrate integration, sensing end formation, and final assembly.

#### 2.3.1. Preparation of the NiCr/NiSi Thermocouple Junction

**Figure 4:**
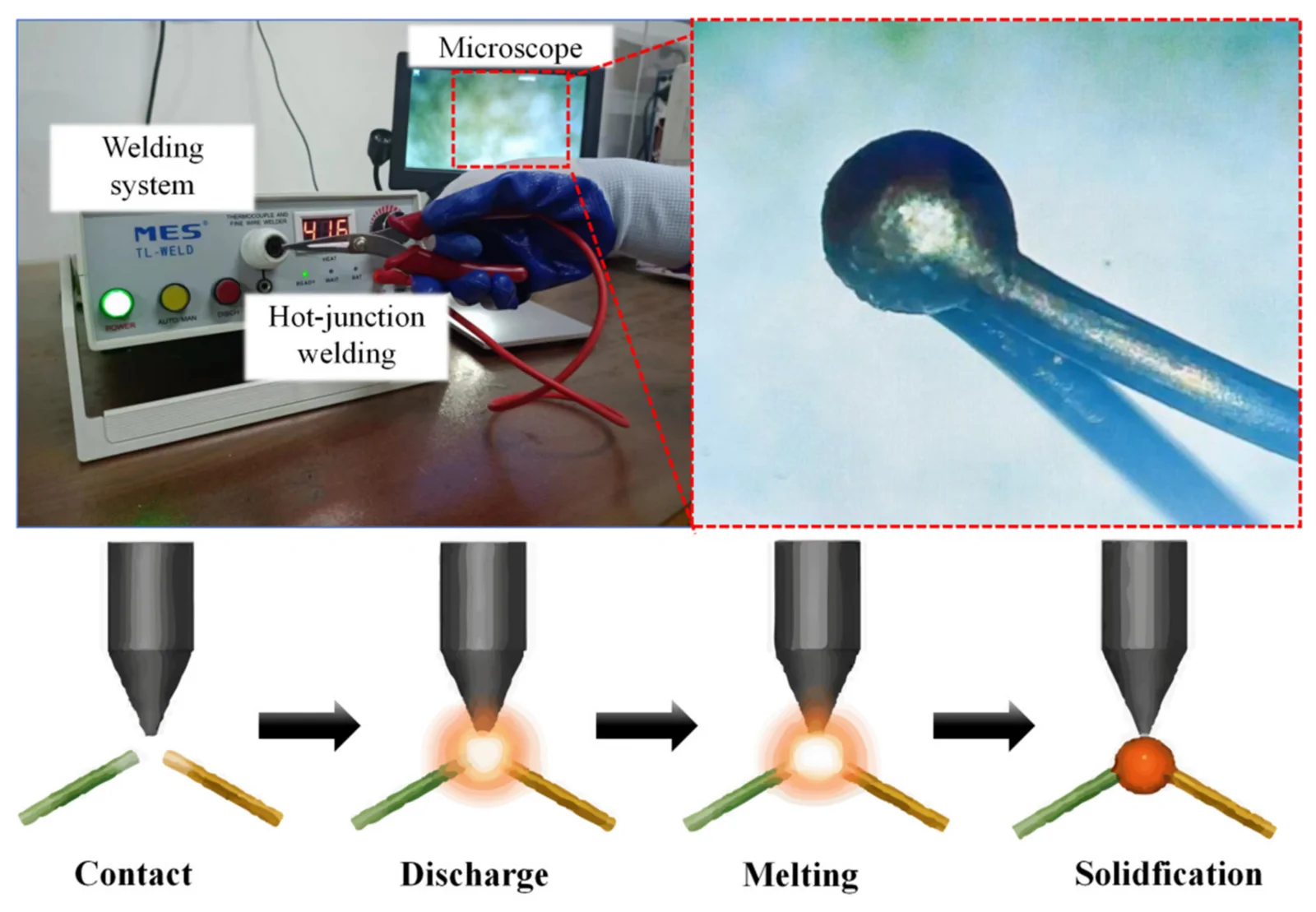
The welding process and enlarged morphology of the fabricated junction.

The thermocouple junction is the active sensing region where the NiCr and NiSi thermoelectrodes form a closed thermoelectric circuit. The quality and stability of the junction directly affect the subsequent integration process and sensing reliability. In this work, the NiCr/NiSi junction was fabricated using a MES TL-WELD thermocouple welding system (Shenzhen Yuanhengtong Technology Co., Ltd., Shenzhen, Guangdong, China), which supports thermoelectrode diameters ranging from 0.1 to 3 mm. Before welding, NiCr and NiSi wires with a diameter of 0.3 mm were ultrasonically cleaned to remove surface contaminants. The two wires were aligned and contacted at their ends to ensure stable electrical and mechanical contact during welding. Subsequently, the thermoelectrodes were fixed in the welding clamps, with approximately 3 mm of the wire ends extending beyond the clamps and contacting the carbon electrode of the welding system. A short-duration welding current was then applied to the contact region, resulting in localized melting of the NiCr and NiSi wires. After solidification, a spherical thermocouple junction was formed at the wire end. The welding process and enlarged morphology of the fabricated junction are shown in Figure 4.

#### 2.3.2. Integration of the Thermocouple Junction into the Al_2_O_3_ Ceramic Substrate

After fabrication of the NiCr/NiSi junction, the thermocouple was integrated into the Al_2_O_3_ ceramic substrate to construct the embedded sensing end. The fabrication process consisted of substrate insulation verification, component cleaning, thermoelectrode insertion, junction positioning, inorganic bonding, thermal treatment, and surface finishing.

**Figure 5:**
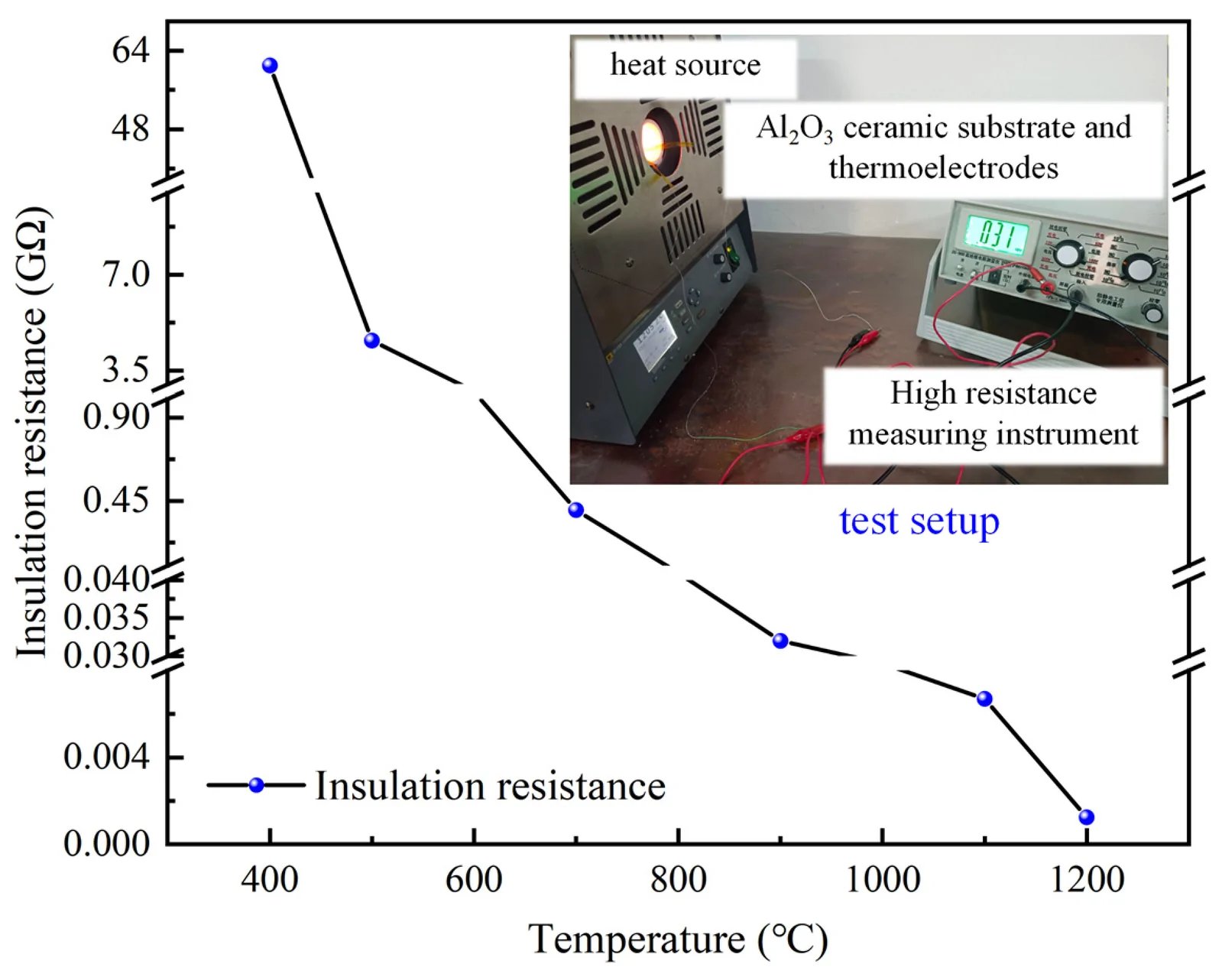
Test of temperature-dependent insulation resistance.

The high-temperature insulation capability of the Al_2_O_3_ ceramic substrate was evaluated. Two metallic wires with the same diameter as the thermoelectrodes (0.3 mm) were inserted into the two independent axial channels of the ceramic substrate, respectively. The two channels were electrically isolated by the ceramic matrix. The insulation resistance between the two channels was measured using a high-resistance insulation tester under elevated temperatures. During the measurement, the furnace temperature was increased from 300 °C to 1200 °C at a heating rate of 5 °C/min. A holding period of 15 min was applied at every 100 °C interval, and the resistance values were recorded after thermal stabilization. The temperature-dependent insulation resistance results are shown in Figure 5. The measurement confirms that the Al_2_O_3_ ceramic substrate maintains electrical isolation between the two thermoelectrode channels throughout the investigated temperature range, providing the electrical foundation for subsequent thermocouple integration.

**Figure 6:**
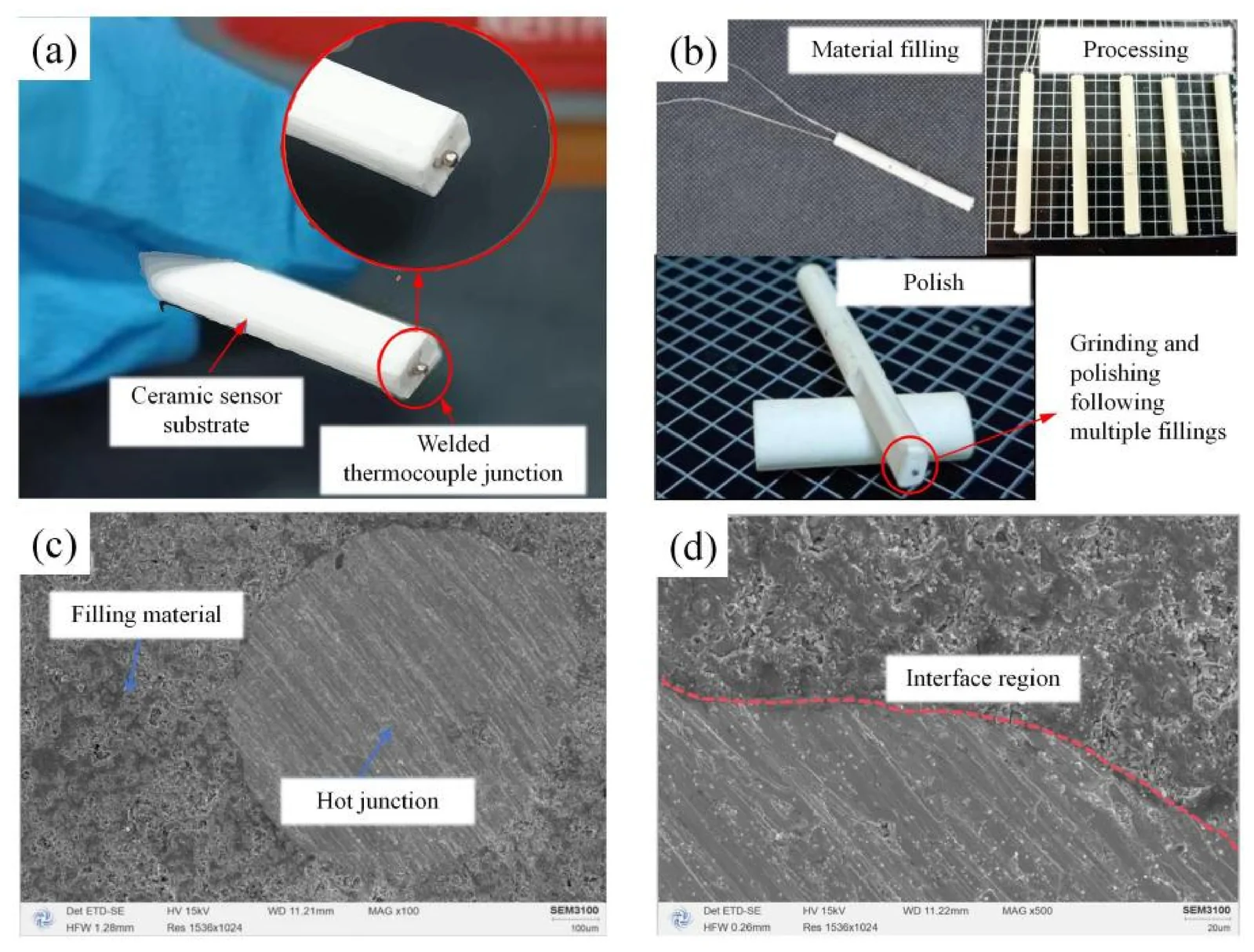
Fabrication process and microstructural characterization of the ceramic embedded junction thermocouple. (**a**) The fabricated ceramic substrate with an embedded NiCr/NiSi thermocouple junction. (**b**) Fabrication procedure of the ceramic embedded junction. (**c**) SEM of the polished hot junction. (**d**) SEM of the interface region between the hot junction and filling material.

Before assembly, both the welded thermocouple junction and the Al_2_O_3_ substrate were ultrasonically cleaned using deionized water, acetone, and absolute ethanol sequentially. Each cleaning step lasted 20 min, followed by drying treatment to remove residual moisture and contaminants. The NiCr and NiSi thermoelectrodes were then inserted separately into the two axial holes with a diameter of 0.5 mm. The welded junction was carefully adjusted and positioned inside the front recess of the ceramic substrate, as illustrated in Figure 6a. This recess provided geometric confinement for the junction and prevented excessive displacement during subsequent processing.

An inorganic bonding material was introduced into the gap between the junction and the ceramic recess. The bonding mixture consisted of inorganic adhesive and high-temperature powder, which was used to establish mechanical integration between the thermocouple junction and the ceramic substrate. During filling, the junction position was carefully maintained to avoid displacement. The filled structure was first cured at room temperature for 2 h and subsequently heat treated at 120 °C for 24 h to enhance the bonding stability. After curing and thermal treatment, the thermocouple junction was mechanically integrated with the ceramic substrate, as shown in Figure 6b. The front surface of the ceramic substrate was subsequently ground and polished to form the final sensing surface. This process served two purposes: improving the surface flatness and controlling the exposed size of the thermocouple junction. During gradual material removal, the junction embedded inside the recess was progressively exposed until it became nearly flush with the ceramic surface.

The effective junction size directly influences the thermal capacity of the sensing region. A larger junction requires more heat absorption to reach thermal equilibrium, resulting in increased thermal inertia and slower temperature response. Therefore, the polishing process was carefully controlled to minimize the exposed junction area while maintaining electrical continuity and structural integrity. Unlike conventional exposed bead thermocouples, the fabricated sensing end retained ceramic support around the junction, allowing the junction to directly interact with the measured environment while reducing mechanical vulnerability.

Ceramic powder was further introduced into the surrounding region and sintered to reinforce the supporting structure. Additional polishing was conducted until a continuous and compact sensing surface was obtained. The prepared sensing surface was cleaned to remove residual particles generated during grinding and polishing and was directly used for SEM characterization. The morphology of the junction region and the surrounding ceramic filling material was examined using a scanning electron microscope (SEM3100, CIQTEK Co., Ltd., Hefei, Anhui, China) equipped with an ETD secondary electron detector. The SEM images were acquired at an accelerating voltage of 15 kV with a working distance of approximately 11.2 mm using the built-in acquisition software of the SEM system. The SEM images of the fabricated sensing end are shown in Figure 6c,d. The SEM images indicate that the thermocouple junction is continuously connected with the surrounding ceramic matrix without obvious defects or cracks, demonstrating successful formation of the ceramic embedded sensing structure. Finally, the rear sections of the axial channels were filled with inorganic bonding material to further stabilize the connection between the thermoelectrodes and the ceramic substrate.

#### 2.3.3. Assembly of the Thermocouple Sensor

**Figure 7:**
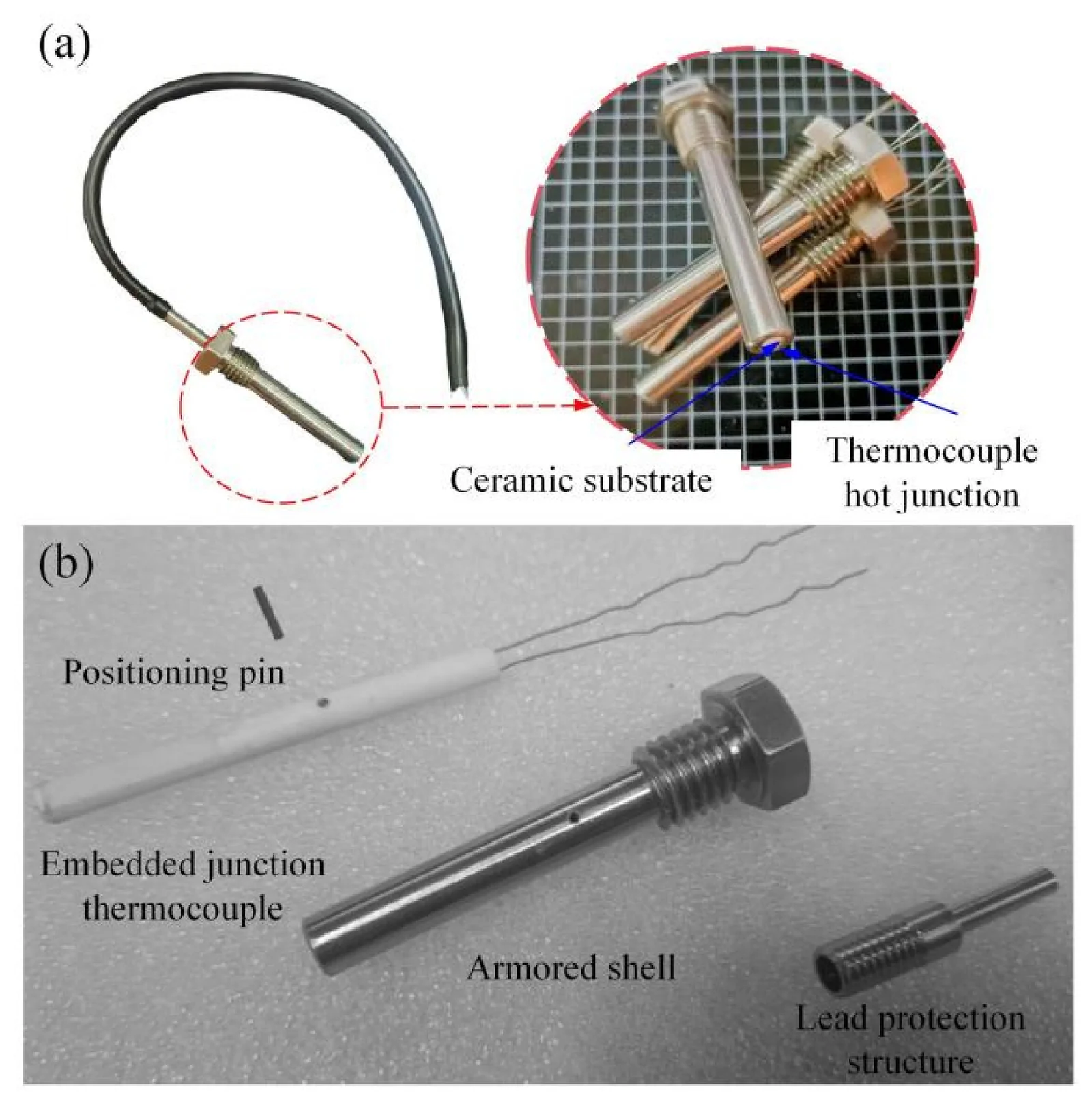
Fabrication and assembly of the ceramic embedded junction thermocouple. (**a**) Photograph of the assembled thermocouple sensor with the nickel alloy armored shell and protected lead substrate. (**b**) Photographs of the components.

After fabrication of the ceramic embedded sensing end, the remaining components were assembled according to the designed structure. The complete sensor consisted of the ceramic sensing component, nickel alloy armored shell, cylindrical pin, rear connector, and lead protection structure, as shown in Figure 7a,b. The ceramic sensing component was first inserted into the nickel alloy armored shell from the front side. The shoulder structure on the ceramic substrate was aligned with the corresponding internal step inside the armored shell, providing axial positioning of the ceramic component and ensuring that the sensing surface remained exposed at the front end of the armored shell. Subsequently, the holes on the ceramic substrate and the armored shell were aligned, and a cylindrical pin with a diameter of 1 mm was inserted to provide additional mechanical fixation. The combined structure of the shoulder, internal step, and cylindrical pin restricted both axial displacement and relative rotation between the ceramic substrate and the armored shell.

The NiCr and NiSi thermoelectrodes were guided through the rear section of the sensor. Insulation sleeves were applied separately to prevent unintended electrical contact between the two thermoelectrodes and between the electrodes and the metallic sheath. The rear connector was then attached to the nickel alloy armored shell through threaded connection, and the lead protection structure was installed to provide additional mechanical protection. Heat shrink tubing was applied at the lead outlet region to improve fixation and insulation of the exposed thermoelectrodes.

## 3. Results and Discussion

After the design and fabrication of the ceramic embedded junction thermocouple, a series of experiments were conducted to evaluate its thermoelectric output characteristics, high-temperature stability, dynamic response, and measurement accuracy. Finally, the sensor was applied in an explosive field test to verify its capability for in situ transient temperature measurement in a confined explosion environment.

### 3.1. Thermoelectric Characteristics and Repeatability

**Figure 8:**
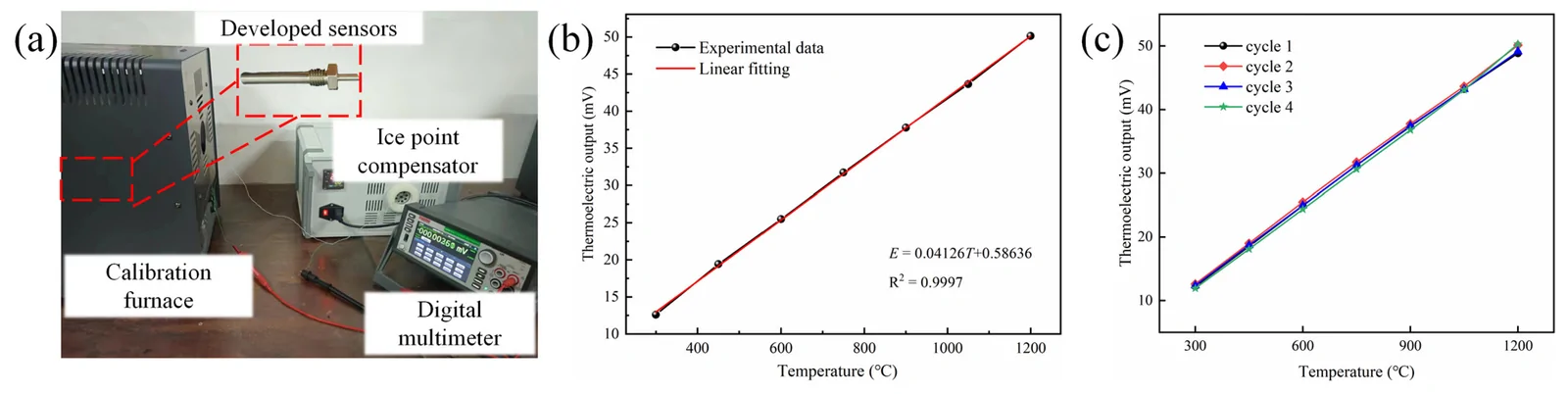
Static thermoelectric calibration and repeatability performance of the ceramic embedded junction thermocouple. (**a**) Static calibration system. (**b**) Relationship between thermoelectric output and temperature. (**c**) Repeated calibration.

The static thermoelectric characteristics of the fabricated sensor were calibrated using a Fluke 9118A high-temperature calibration furnace (Fluke Calibration, Everett, WA, USA). During calibration, the fabricated thermocouple and a standard K-type thermocouple were simultaneously placed in the uniform temperature region of the furnace. The output terminals of the sensor were connected to a 6190A electronic ice point compensator through compensation wires, and the thermoelectric voltage was recorded using a Tektronix DMM7510 digital multimeter (Fluke Calibration, Everett, WA, USA). The calibration temperature range was set from 300 to 1200 °C. Starting from 300 °C, measurements were performed at intervals of 150 °C after thermal stabilization at each temperature point. The calibration system and measured thermoelectric voltage temperature relationship are shown in Figure 8a,b.

Within the temperature range of 300–1200 °C, the output voltage E exhibited an approximately linear relationship with the measured temperature T. The calibration curve was fitted using the least squares method:

(3) E=0.04126T+0.58636

The fitting slope was 0.04126 mV/°C, corresponding to a Seebeck coefficient of 41.26 μV/°C. The coefficient of determination R^2^ reached 0.9997, indicating excellent linearity between the thermoelectric output and temperature. The results demonstrate that the integration of the thermocouple junction into the Al_2_O_3_ ceramic substrate did not significantly affect the thermoelectric conversion behavior. The embedded junction maintained a stable thermoelectric relationship over the investigated temperature range.

To evaluate the output consistency after repeated thermal cycling, four independent calibration cycles were performed. Each cycle consisted of heating from 300 to 1200 °C followed by cooling back to room temperature. The calibration curves obtained from four cycles are shown in Figure 8c. As can be seen from Figure 8c, the curves from the four calibrations are fairly consistent, indicating good repeatability.

The repeatability was evaluated using the standard deviation of the measured values:

(4) $$\sigma =\sqrt{\frac{\sum_{i=1}^{n}{\left(x_{i}-\bar{x}\right)}^{2}}{n-1}}$$

where n represents the number of measurements, $x_{i}$ is the measured value of each cycle, and $\bar{x}$ is the average value of the repeated measurements.

The repeatability error rate was defined as:

(5) $$\delta =\frac{\sigma }{x}\times 100\%$$

where δ represents the repeatability error rate. The data from the four calibrations and the calculated repeatability errors are recorded in Table 3. It can be seen from the data in the table that the maximum repeatability error calculated from the four calibration cycles was 2.32%. The repeated calibration curves exhibited highly consistent trends over the entire temperature range, indicating that the fabricated sensor maintained stable thermoelectric output after repeated thermal cycling. This result demonstrates that the ceramic embedded junction structure, including the NiCr/NiSi junction, inorganic bonding layer, and Al_2_O_3_ substrate, possessed sufficient thermal stability during repeated heating and cooling processes.

### 3.2. High-Temperature Performance

The high-temperature stability of the fabricated ceramic embedded junction thermocouple was evaluated using a Fluke 9118A high-temperature calibration furnace. The same signal acquisition system used for static calibration was employed to record the thermoelectric output during high-temperature exposure. Three types of thermal loading conditions were designed, including repeated thermal cycling at 1200 °C, continuous exposure at 1200 °C, and stepwise temperature variation from 300 to 1200 °C.

**Figure 9:**
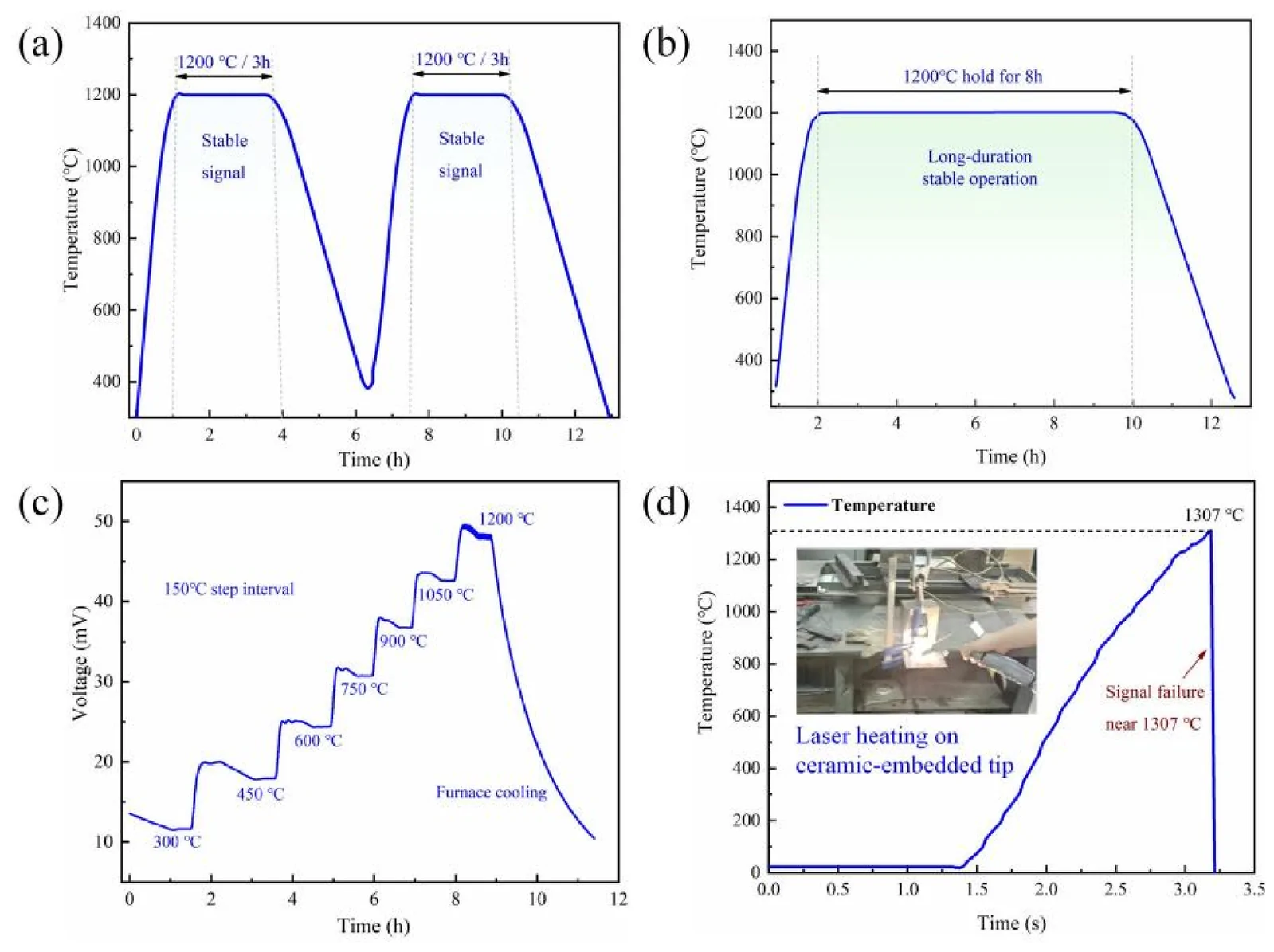
High-temperature performance evaluation of the ceramic embedded junction thermocouple. (**a**) Repeated thermal cycling. (**b**) Long-term continuous operation. (**c**) Thermoelectric output response under stepwise temperature variation. (**d**) Local laser-heating-induced temperature response and signal-failure behavior.

For the thermal cycling test, the sensor was heated from 300 to 1200 °C and maintained for 3 h, followed by cooling to 300 °C. This heating and cooling process was repeated twice, and the corresponding thermoelectric output curves are shown in Figure 9a. During both thermal cycles, the sensor maintained continuous thermoelectric output throughout the heating, holding, and cooling stages without signal interruption. The consistent output behavior indicates that repeated thermal expansion and contraction did not cause electrical discontinuity within the NiCr/NiSi junction, inorganic bonding region, or electrode connection area.

To further evaluate the long-term thermal stability, the sensor was continuously exposed to 1200 °C for 8 h, as shown in Figure 9b. During the entire high-temperature holding period, the thermoelectric signal remained continuously detectable without obvious interruption or abnormal fluctuation. Compared with short duration heating, this test provides a more direct evaluation of the stability of the embedded junction structure under prolonged thermal exposure. The result suggests that the ceramic embedded junction and its supporting structure can maintain stable thermoelectric output during extended operation at elevated temperature.

In addition, a stepwise temperature loading test was conducted to investigate the output response under multiple temperature levels. Eight temperature points from 300 to 1200 °C were selected with an interval of 150 °C, and each temperature stage was maintained for 20 min before increasing to the next level. After reaching 1200 °C, the sensor was naturally cooled with the furnace. As shown in Figure 9c, the thermoelectric output increased progressively with increasing furnace temperature, and stable voltage signals were obtained at each temperature plateau. During the cooling process, the output voltage decreased correspondingly without significant deviation from the heating trend. These results demonstrate that the embedded junction structure maintained a stable thermoelectric conversion relationship over a wide temperature range.

The above results indicate that the ceramic embedded junction design provides sufficient thermal stability for high-temperature measurements. The combination of the Al_2_O_3_ ceramic substrate, inorganic bonding layer, and mechanical constraint structure enables the thermocouple junction to maintain electrical continuity under repeated heating, long duration exposure, and temperature-variation conditions. Meanwhile, the direct thermal contact between the exposed junction region and the measured environment was preserved, ensuring continuous temperature response after high-temperature treatment.

To further investigate the failure behavior of the sensor under localized high-temperature loading, a localized laser heating experiment was performed using a C200T-SH2000 handheld laser welding system (Zhejiang Junyi Laser Equipment Co., Ltd., Ningbo, Zhejiang, China). The laser beam was focused directly onto the sensing end, and the transient thermoelectric output was recorded using the DMM7510 multimeter. The temperature response curves are shown in Figure 9d. As shown in Figure 9d, the output voltage continuously increased with increasing laser heating intensity. When the recorded temperature approached approximately 1307 °C, the thermoelectric signal suddenly disappeared, indicating the failure of the sensor under the applied localized heating condition. Compared with the furnace tests, the laser heating produced a more concentrated and rapidly changing thermal load. The result therefore provides further evidence of the high-temperature tolerance of the ceramic embedded junction structure under rapid local heating, complementing the continuous operation demonstrated at 1200 °C in the furnace test.

### 3.3. Dynamic Response Characteristics

**Figure 10:**
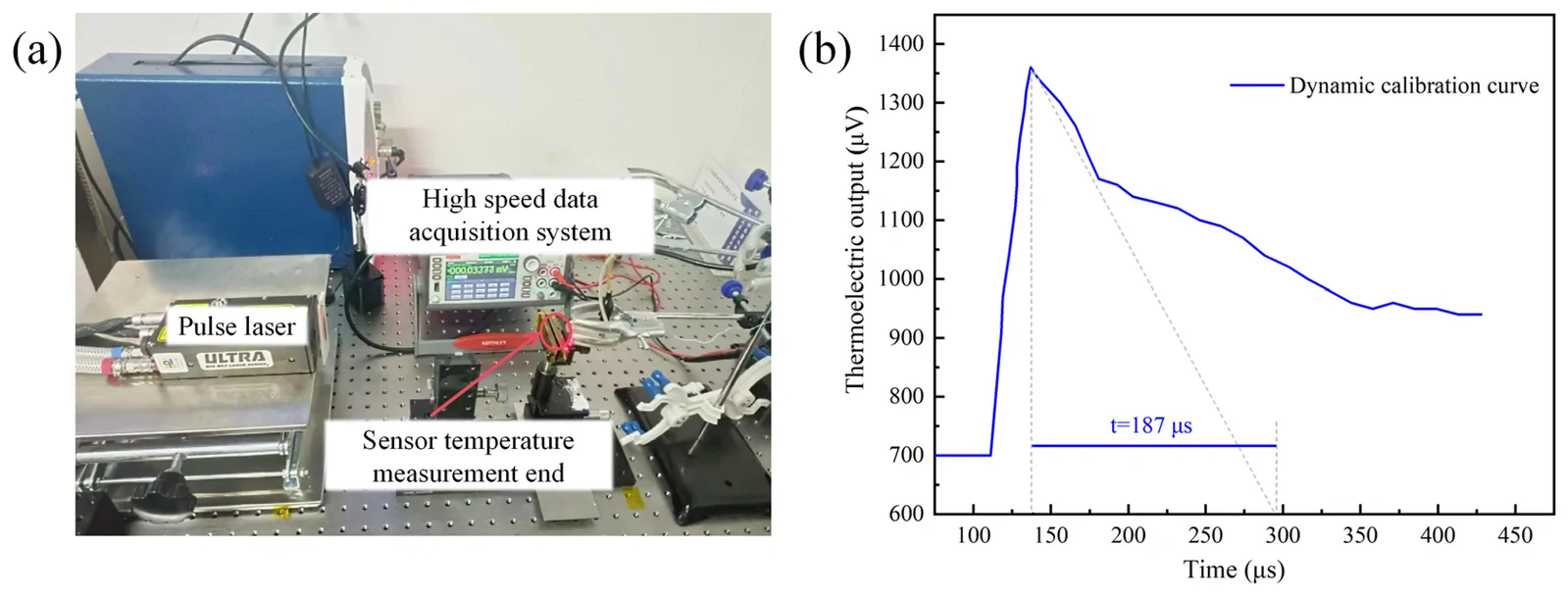
Pulsed laser dynamic calibration of the ceramic embedded junction thermocouple. (**a**) Experimental configuration for dynamic response measurement. (**b**) Dynamic calibration curve.

The transient temperature variation in an explosion environment occurs within a short time scale, and therefore the dynamic response capability of the thermocouple directly determines the accuracy of transient temperature acquisition. To evaluate the response behavior of the fabricated ceramic embedded junction thermocouple, a pulse laser heating method was employed to characterize its dynamic response. The dynamic calibration system consisted of a pulse laser source, laser control unit, sensor fixture, and high-speed data acquisition system, as shown in Figure 10a. During the measurement, the sensor was fixed on a supporting platform, and the laser beam was accurately focused on the exposed thermocouple junction at the sensing end. A short duration laser pulse was applied to generate a rapid thermal excitation on the junction, resulting in a transient thermoelectric voltage response. After the laser pulse was removed, the generated heat gradually dissipated through the surrounding ceramic substrate and metal structure, and the output signal returned toward the initial state. The complete transient voltage response process was recorded for dynamic response analysis.

The measured transient response curve is presented in Figure 10b. According to the characteristic response obtained from the pulse excitation curve, the response time of the fabricated sensor was approximately 187 μs, indicating a sub millisecond response capability. This result demonstrates that the ceramic embedded junction structure can still achieve rapid thermal response despite the introduction of a supporting ceramic substrate.

The rapid response of the developed thermocouple is related to the structural design of the sensing end. Previous studies have demonstrated that reducing the thermal mass of the sensing element is an effective approach for improving transient response. Thin film thermocouples with small sensing regions can achieve rapid temperature response due to their reduced thermal inertia [24]. However, temperature measurements in confined explosive environments require a balance between response speed and structural stability, as thinner sensing elements may experience reduced mechanical robustness under severe loading conditions [26].

Based on these considerations, the ceramic embedded junction configuration was adopted in this work. The NiCr/NiSi junction was embedded within the Al_2_O_3_ ceramic substrate, while a limited junction region was exposed at the sensing face after grinding and polishing. The exposed junction enables direct heat exchange with the measured environment, whereas the surrounding ceramic provides mechanical support and electrical insulation. This configuration reduces the effective thermal mass of the active sensing region while maintaining the structural stability required for transient temperature measurements in confined explosive environments.

Compared with conventional thermocouples with larger sensing junctions, the reduced effective junction size provides improved capability for tracking rapid temperature variations. Meanwhile, unlike fully exposed fine-wire thermocouples, the embedded configuration provides additional structural support for the junction, which is beneficial for maintaining sensor integrity under severe environments involving impact and high temperature. These results indicate that the proposed ceramic embedded junction design provides a balanced solution between rapid thermal response and structural stability, offering potential for transient temperature measurements in confined explosion environments.

### 3.4. Calibration Accuracy and Uncertainty Evaluation

**Figure 11:**
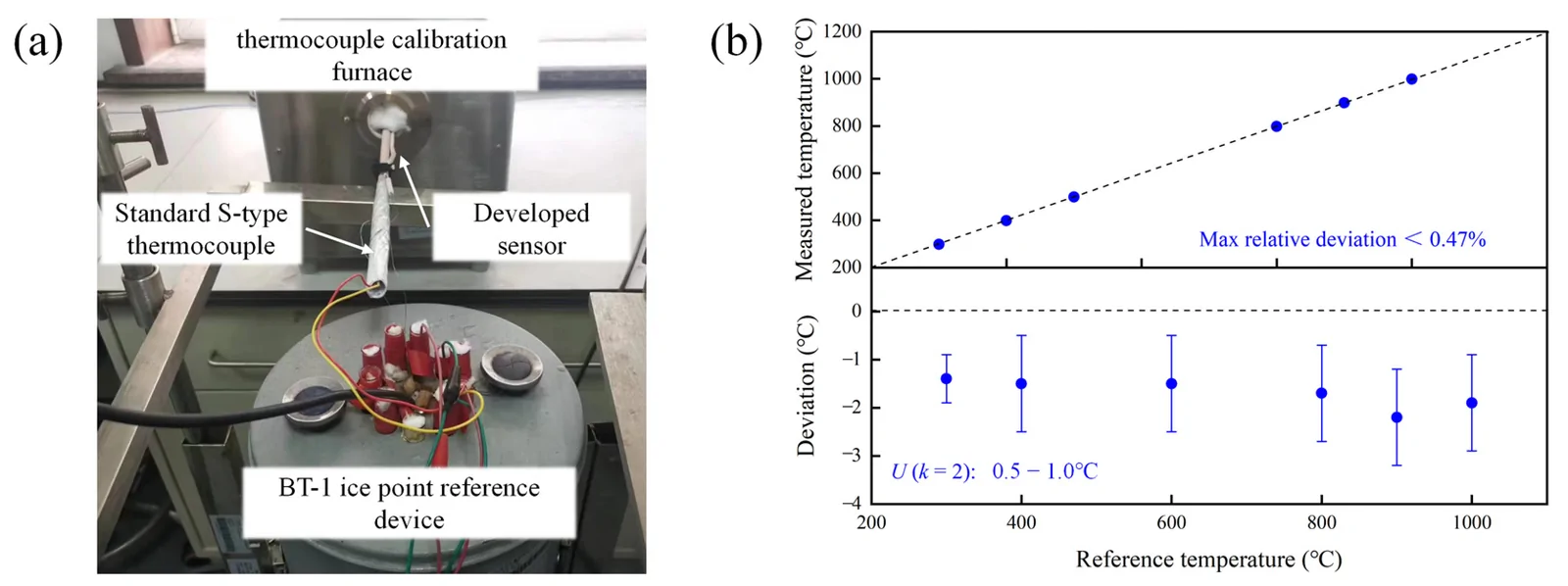
Calibration accuracy evaluation of the ceramic embedded junction thermocouple. (**a**) Standard temperature calibration system. (**b**) Relevant errors and expanded uncertainty.

To evaluate the measurement accuracy of the fabricated ceramic embedded junction thermocouple, an independent calibration procedure was performed using a standard temperature calibration system, as shown in Figure 11a. The calibration was conducted using a KRJ-600 thermocouple calibration furnace (Taian Panran Measurement and Control Technology Co., Ltd., Tai’an, Shandong, China), a BT-1 ice point reference device, a precision digital multimeter, and a standard S-type thermocouple as the reference sensor. During the calibration process, the fabricated thermocouple and the reference S-type thermocouple were simultaneously positioned in the same temperature zone of the calibration furnace. The thermoelectric output of the fabricated sensor was recorded at each calibration point, and the corresponding temperature values were obtained from the reference thermocouple. The calibration procedure was performed at six temperature points, including 300, 400, 500, 800, 900, and 1000 °C.

The temperature deviation between the fabricated sensor and the reference thermocouple was calculated, and the expanded uncertainty was evaluated with a coverage factor of k = 2. As summarized in Table 4 and Figure 11b, the measured temperature deviations ranged from −1.4 to −2.2 °C, corresponding to a maximum relative deviation of 0.47%. The expanded uncertainty was within 0.5–1.0 °C over the investigated temperature range. The calibration results confirm that the fabricated sensor maintains reliable temperature conversion characteristics after integration of the thermocouple junction into the Al_2_O_3_ ceramic substrate. The small deviation from the reference temperature indicates that the ceramic embedded junction structure does not introduce significant measurement error, while the exposed sensing region formed after surface polishing preserves effective thermal interaction with the measured environment.

### 3.5. In Situ Transient Temperature Measurement in an Explosive Field

**Figure 12:**
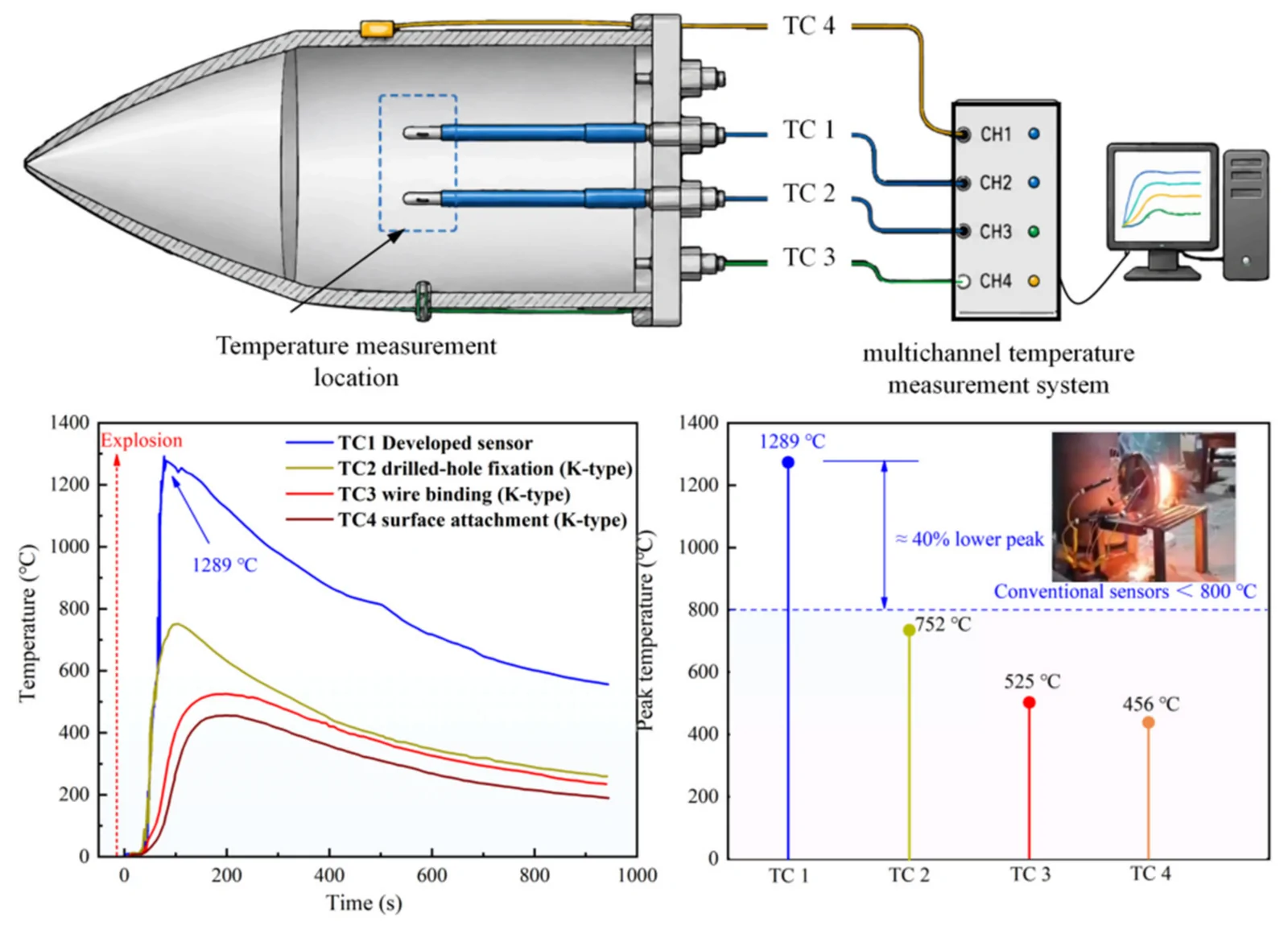
In situ transient temperature measurement in a confined explosive field.

After the laboratory characterization, the fabricated ceramic embedded junction thermocouple was further applied in a confined explosive environment to evaluate its practical capability for in situ temperature measurement. The experiment was conducted inside the thermal nose cavity of a target projectile under a static explosion condition. The purpose of this test was to verify whether the fabricated sensor could maintain stable installation, signal transmission, and temperature acquisition capability under a real explosive environment. Four thermocouples were installed inside the thermal nose cavity during the experiment. Among them, one was the fabricated ceramic embedded junction thermocouple (TC 1), while the other three were conventional K-type thermocouples used for comparison. The conventional thermocouples were installed using different approaches, including drilled-hole fixation (TC 2), wire binding (TC 3), and surface attachment (TC 4). All sensor outputs were simultaneously collected using a Fluke 1586A high precision multichannel temperature measurement system (Fluke Calibration, Everett, WA, USA). The sensor arrangement and the measured temperature curves are presented in Figure 12.

Following explosion initiation, all thermocouples recorded a clear temperature increase followed by a gradual cooling process. As shown in Figure 12, the fabricated sensor exhibited a faster temperature rise compared with the conventional K-type thermocouples and captured a higher temperature peak during the thermal evolution process. The maximum temperature recorded by the developed sensor was approximately 1289 °C, occurring about 80 s after explosion initiation. In comparison, the maximum temperatures recorded by the three conventional K-type thermocouples were below 800 °C, which was approximately 40% lower than the peak temperature of 1289 °C measured by the fabricated sensor. The temperature response of each thermocouple was influenced by its sensing structure, dynamic response, and the local thermal coupling associated with its installation configuration. The ceramic embedded junction provides a reduced effective sensing region, while maintaining direct thermal interaction between the junction and the surrounding environment. Together with the sub-millisecond response demonstrated by the pulse laser calibration, these characteristics improve the capability of the developed sensor to follow rapid temperature variations. The different installation methods of the conventional thermocouples may also result in differences in local thermal contact and heat transfer at the measurement position.

During the entire measurement period exceeding 16 min, the fabricated sensor maintained continuous signal output without apparent interruption. The successful operation in the confined explosive environment demonstrates that the ceramic embedded junction thermocouple can achieve reliable temperature acquisition after practical installation. These results verify the feasibility of the proposed sensor for localized temperature measurements in confined explosion fields.

## 4. Conclusions

A ceramic embedded junction thermocouple was developed for localized transient temperature measurement in confined explosive environments. By integrating a NiCr/NiSi thermocouple junction into an Al_2_O_3_ ceramic substrate and designing an armored insertion probe, the proposed sensor achieved a balance between junction protection and rapid thermal response. The fabrication process and performance evaluation demonstrated the feasibility of applying the proposed structure for harsh high-temperature measurement. The sensor structure was designed based on the requirements of confined explosive environment measurement. Finite element analysis was employed to optimize the overall dimensions and structural parameters of the probe, providing the basis for the selection of the ceramic substrate and armored configuration. The ceramic embedded junction structure was subsequently fabricated through thermocouple welding, inorganic bonding, thermal treatment, and surface grinding. The Al_2_O_3_ ceramic substrate provided electrical insulation and local mechanical support for the NiCr/NiSi thermocouple junction, while the controlled exposed sensing region maintained direct thermal interaction with the measured environment. The fabricated sensor exhibited stable thermoelectric characteristics, with a Seebeck coefficient of 41.26 μV/°C and a linear fitting coefficient of determination of 0.9997 in the range of 300–1200 °C. The sensor operated continuously at 1200 °C for 8 h without signal interruption.

The dynamic and measurement performance of the sensor were further verified. Pulse laser calibration showed a characteristic response time of approximately 187 μs, indicating the sub-millisecond response capability of the embedded junction structure. The independent calibration results showed a maximum relative deviation below 0.47% within 300–1000 °C. In the confined explosive environment, the developed sensor successfully recorded the post-explosion temperature evolution and measured a peak temperature of approximately 1289 °C, whereas conventional K-type thermocouples with different installation configurations recorded peak temperatures below 800 °C. These results demonstrate that the proposed ceramic embedded junction thermocouple provides an effective approach for localized transient temperature measurement in confined explosive environments by integrating structural optimization, junction protection, rapid response, and reliable temperature-sensing capability.

## Figures and Tables

**Table 1:** Numerical results of fluid–structure coupling simulations for sensors with different outer diameters.

Air Velocity (m/s)	Structural Response	Sensor Outer Diameter (mm)				
		4	6	8	10	12
0.5	Maximum deformation (mm)	0.00043	0.00012	0.00008	0.00003	0.00001
	Equivalent stress (MPa)	0.1585	0.0809	0.0433	0.0275	0.0259
1	Maximum deformation (mm)	0.00173	0.00051	0.00024	0.00012	0.00007
	Equivalent stress (MPa)	0.6351	0.3234	0.1733	0.1108	0.103
1.5	Maximum deformation (mm)	0.00393	0.00116	0.00054	0.00029	0.00014
	Equivalent stress (MPa)	1.4415	0.7303	0.3941	0.2510	0.1479

**Table 2:** Thermophysical and mechanical properties of materials used in the sensor simulation [32,33].

Material	Thermal Conductivity W/(m.k)	Specific Heat Capacity J/(kg.k)	Density kg/m^3^	Young’s Modulus GPa	Poisson’s Ratio
NiCr	14.9	460	8400	140	0.3
NiSi	11.2	450	8400	200	0.3
Al_2_O_3_	30	900	3950	380	0.22
Nickel alloy	11.5–17.3	420–460	8000–9000	190–220	0.3–0.33

**Table 3:** Repeatability experimental data.

Temperature/°C	Cycle 1/mV	Cycle 2/mV	Cycle 3/mV	Cycle 4/mV	Repeatability Error/%
300	12.21	12.60	12.36	11.92	2.32
450	18.52	19.01	18.69	18.09	2.06
600	24.91	25.48	24.94	24.35	1.85
750	31.21	31.72	31.26	30.61	1.46
900	37.33	37.77	37.41	36.77	1.11
1050	43.21	43.64	43.19	43.10	0.56
1200	48.84	50.12	49.13	50.32	1.47

**Table 4:** Calibration results and measurement uncertainty at different reference temperatures.

Reference Temperature/°C	Deviation/°C	Relative Deviation	Expanded Uncertainty/°C (k = 2)
300	−1.4	0.47%	0.5
400	−1.5	0.38%	1.0
500	−1.5	0.30%	1.0
800	−1.7	0.21%	1.0
900	−2.2	0.24%	1.0
1000	−1.9	0.19%	1.0

## Data Availability

The data presented in this study are available on request from the corresponding author.
